# The Virtuous Interplay of Infrastructure Development and the Complexity of Nations

**DOI:** 10.3390/e20100761

**Published:** 2018-10-03

**Authors:** Matthieu Cristelli, Andrea Tacchella, Masud Cader

**Affiliations:** 1 Country Analytics, International Finance Corporation–World Bank Group, Washington, DC 20433, USA; 2 Institute of Complex Systems, National Research Council (CNR), 00185 Rome, Italy

**Keywords:** economic complexity, infrastructure, economic development

## Abstract

Does the infrastructure stock catalyze the development of new capabilities and ultimately of new products or vice-versa? Here we want to quantify the interplay between these two dimensions from a temporal dynamics perspective and, namely, to address whether the interaction occurs predominantly in a specific direction. We therefore need to measure the complexity of an economy (i.e., its capability stock) and the infrastructure stock of a country. For the former, we leverage a previously proposed metrics, the Economic Fitness (Tacchella, A.; et al. *Sci. Rep.* **2012**, *2*, 723). For the latter, we propose a new purely statistical indicator which is the *principal component* performed on the 47 infrastructure indicators published by the World Bank. The proposed indicator still belongs to the class of linear combination of relevant indicators but, differently from standard economic indicators of the same type as the *Connectivity Index*, the *HDI*, etc, the weights of the linear combination are not subjectively chosen or re-calibrated on a regular basis but they are those which capture the highest fraction of the information encoded in the initial dataset. The two metrics allow the study of the dynamics in the *Economic Fitness-Infrastructure* plane and reveal the existence of two regimes: one for low Fitness where the infrastructure and the complexity of an economy are unrelated and a second regime where the two dimensions are tightly related. To quantify the interplay of the two dimensions in this latter regime, we assume a parsimonious linear dynamic model and the emerging picture is that: (i) the feedback occurs in both directions; (ii) on the short-term (<3 years) the predominant direction of interaction is from infrastructure to capability stock; (iii) while for longer time scale (>3 years) the interaction is reversed, new capabilities lead to increasing infrastructure stock.

## 1. Introduction

The stock of infrastructures intuitively plays an important role in the development trajectory of countries. It is easy to spot commonalities in all developed countries and emerging countries catching up the developed economies in terms of available infrastructures: extended airport and railway networks, road networks, communication networks, stable energy production/access, easily accessible freshwater, services to enable easier commercial exchanges, etc.

On the other hand, countries below the poverty threshold tend to have these dimensions significantly less developed or even missing. In this perspective, infrastructure appears as being necessary ingredients for the economic development. However, the opposite argument has the same economic soundness as the previous one: the further development of infrastructures needs the achievement of a stable enough and sustained growth (in other words, the development of institutions which enable stable growth and allow for the development of infrastructures). It is indeed hard to imagine the development of extended and effective communication systems in underdeveloped countries or in regions undergoing instabilities or even conflicts.

The most likely scenario is that both arguments are at work and the two dynamically interact (not necessarily in a symmetric fashion as we will see): i.e., the increase of complexity of an economy may trigger the development of infrastructure and vice-versa, new infrastructure may enable more complex production.

The importance of quantifying the interplay between the two dimensions is particularly relevant in the formulation of development policies to design effective plans to foster (or ignite) growth. Diagnosing the relationship and the different phases of the transmission links between growth, complexity of the output of an economy and infrastructures stocks is critical to guide the investments in the most appropriate sector with the most effective timing. Let us suppose we find that an increasing complexity of the capability stock of the economic output triggers the infrastructure sprout and the reversed action is instead marginal or that in some regimes (e.g., developed countries) some types of infrastructure affect economic development. In such a scenario an optimal funneling of resources would then be oriented towards the acquisition of new capabilities rather than to the direct development of new infrastructures.

The literature on infrastructures is wide and witnesses the importance of quantifying the previously mentioned transmission links. However, despite three decades of researches since the seminal work of Aschauer [1], the links and the role of infrastructures for growth is still an open and debated question. While there is a general consensus on the fact that infrastructures matter for growth, we are far from having a consensus on how, how much and when they matter.

A first difficulty already arises when we try to measure and quantify the infrastructure stock of an economy. A direct assessment is often elusive as witnessed by a lack of consensus on how to effectively measure this stock in a synthetic way.

Several works have leveraged indirect and aggregate proxies such as the public capital finding a positive relationship between public investment in infrastructures and growth [2,3]. However, it has been shown in [4] that leveraging only public inflows underestimates the real impact of infrastructures since the private contribution is left out in such analyses. In addition, the heterogeneity in the efficiency of the transmission chain from capital inflows to final impact on the economic system may affect the robustness of these approaches.

With a focus on the measurement aspect, other approaches try a more granular analysis by linearly combining variables or pillars related to infrastructures as indicators of water, energy supply, road and communication networks, etc. The most known *meta* indicators following this strategy are the *Connectivity Index*, *Human Development Index, (HDI)*, *GCI Infrastructure pillar*. However, as discussed in the following, these indicators often rely on survey based variables and/or on subjective/region dependent weighting schemes to define the indicator.

Turning our attention on the impact of infrastructures on the economic development, three decades of literature have produced several results but findings are often non-completely conclusive or not coherent across different works. The main results are about:

- **the relation between infrastructures and different stages of development**: in [5,6] it is found that there is no clear relationship with the stage and the impact seems to be independent on the maturity of an economy, while in [7,8] the authors lean towards a dependence of the impact of infrastructures on the level of development;
- **the impact of specific sectors**: (i) energy [7]; (ii) water supply and sanitation [9,10]; (iii) communication networks [11,12]; (iv) road networks [13]; (v) port facilities [14], etc. All works find a general positive impact of all the sectors on growth although some of them find conclusive results only on specific regions or periods.

We refer to [15] for a detailed review of the field.

This work contributes to the existing literature along the following axes:

1. We propose a new strategy to quantify the infrastructure stock of a country. We propose a broad statistical indicator issuing from a *principal component analysis* (PCA, [16,17]) applied to the 47 infrastructure indicators made publicly available by the World Bank Data portal [18]. The proposed indicator still belongs to the family of linear combination of economic variables as the previously cited GCI index, HDI, etc but the weights are chosen on a statistical and data-driven basis. The PCA is in fact a commonly and extremely effective statistical technique of dimension reduction to define low dimensional spaces (in our case a one dimensional indicator) carrying a significant fraction of the information (in the jargon of PCA, a significant fraction of the variance of the data) encoded in the original large set of variables (in this case the 47 indicators we start from).
2. We want to quantify the dynamic interplay between infrastructure stock and the economic development of nations and ultimately their capability stock in order to assess:
  (a) in which regime the interplay is active;
  (b) whether there exists a preferred direction in this interaction, namely, *roughly speaking*, does the level of economic complexity *cause* the development of infrastructure more than the opposite interaction (we are intentionally misusing the term *cause* but it qualitatively provides the spirit of this work)?
  (c) whether the interplay depends on the time horizon we look at.
  It is worth noticing that standard works typically focus only on the impact of infrastructures on development and growth, here we want to assess the impact in both directions because we believe that a complete picture to understand the role of the infrastructure can be obtained only within a framework allowing for the feedback to be dynamic and in both ways. To achieve this, we embed in a dynamical system approach our new indicator for the infrastructure stock and a metrics for the capability stock similarly to what has been proposed for growth in [19,20,21]. This leads to study the features of the interplay in the plane defined by these two dimensions and model their feedback as a parsimonious pair of equations depending linearly on the past lagged values of the two metrics and eventually assessing the statistical significance of the coefficients. Such a modeling of the interplay corresponds to test the *Granger* causality [22] between the two dimensions in the two possible ways. As a metrics for the capability stock of a nation, we leverage an already existing measure, the Economic Fitness, [23,24,25] which has been recently included in the World Bank datasets as one of the development indicators [26].
3. We investigate whether the infrastructure stocks depend on the specific phase of development of a country.
4. We show how to extend the general ideas grounding the Economic Complexity approach [24] beyond its initial scope to new dimensions as represented by infrastructure quantifying the tight and dynamic linkage between economic development and infrastructures.

The main findings of this work are:

1. the principal component, i.e., our proposed indicator, carries more than one third of the total information encoded in the 47 indicators witnessing that this direction is highly non-trivial. Almost all the loadings on the original variables are positive (or negative but in these cases the variable has a negative sense), this allows for a direct economic interpretability of our indicator;
2. by analyzing the dynamics in the plane defined by the Economic Fitness and our infrastructure indicator, we find that the interplay exists in both directions and there are mainly two regimes driven by the level of complexity of the capability stock of a nation:
  (a) when the capability stock is low, i.e., low Fitness countries, the infrastructure stock is significantly below the cross-section mean, uncorrelated with the level of Fitness and testing for the *Granger* causality does not provide any conclusive results;
  (b) above a specific threshold of complexity of the capability stock (log(Fitness)>−2), infrastructures and Fitness get correlated and related by a linear upward relation.
3. although both directions are active above the threshold log(Fitness)=−2, the dominant direction of the interplay results to depend on the time horizon of the lagged interaction we consider in the adopted model of interplay: when we include a dependence on few lagged past values (up to 2 years), the infrastructures *Granger* cause more the Fitness than the opposite relation, while when more lags are included (>3 years), the interplay is generally more significant and the dominant way is reversed, the leading variable turns to be the Economic Fitness. In all cases, the interaction is proactive in both directions as the coefficients of the interactions are predominantly positive. This seems to suggest that, on the short term, the infrastructure stock drives the acquisition of new capabilities but, on longer time horizon, an increasing capability stock is the driving force guiding the infrastructure stock.
4. the loadings of the principal component performed on countries conditionally on the level of Economic Fitness show some differences. However, further analyses are required to understand whether these differences are significative. At this stage, we know for sure that there is a clear dependence of the intensity of the infrastructure stock on the capability stock but whether the stock itself changes is still an open question.

The paper is organized as follows: in section *Material and Methods* we briefly review the Economic Fitness metric and we introduce the proposed metrics for infrastructures issuing from the PCA and we discuss the grounding ideas of this approach. In section *Results*, we discuss our main findings while in the last section we provide conclusions and perspectives.

## 2. Material and Methods

### 2.1. Measuring the Complexity of an Economy: The Economic Fitness of a Country

To measure the level of complexity of an economic system (i.e., its capability stock), we use a recently proposed metrics of country diversification, the Economic Fitness [24]. The Economic Fitness of a country is essentially a measure of diversity of an economy weighted by the complexity of the products a country is able to produce on a competitive basis. The metric is the result of a non-linear iterative procedure defined on the bipartite network countries-exported goods which is made accessible via the UN Comtrade dataset [27,28]—see also Appendix A for details on the datasets. The general spirit of the metrics is:

- the more a country is diversified and the more is able to produce/export complex goods, the higher will be its Economic Fitness;
- the more exclusive a product and the less poorly fit countries are able to export it, the more complex it will be.

We refer to [23,24] for a detailed description of the mathematical specifications and to [23,29,30,31,32] for a discussion about the novelty and the advantages of this metric with respect to previous attempts in the literature [33]. In the present paper, we use the original definition of the metrics which leverages only UN Comtrade data. Recent attempts in [34] and especially in [35] have proposed to extend the metrics including also the export of services. However, here we prefer to stick to the original version without services for two reasons:

- the metrics with and without services tend to be very correlated;
- the boundaries between services and infrastructures are often blurred and therefore we prefer not to include services in our measure of Economic Complexity in order to keep the two dimensions as separated as possible.

The Economic Fitness has been shown to be highly informative and predictive for the potential of growth of countries when coupled with a metrics of wealth of a nation as the gross domestic product *per capita* (hereinafter GDPpc) [19,20,21,36]. This coupled analysis enables to underpin different regimes of growth: regimes where the growth of a country tends to unpredictable and regimes where the growth is stabler and more predictable. We will be able to build on these results because we will study the dynamics of the infrastructures in relation with the Economic Fitness which will allow us to interpret our findings within a wider framework.

### 2.2. Measuring the Level of Infrastructures: A Statistical Approach

In order to measure the infrastructure, we want to develop an approach providing a synthetic indicator as in the case of the Economic Fitness. In the case of Economic Fitness, we successfully encapsulated all the endowments (also named capabilities in the literature [37]) of a country into a single figure because we can directly access an economic level which (i) is highly standardized across countries for custom/tax reasons; (ii) is the final result of the complex interaction of countries’ endowments. This level is the final output of an economy: what a country is able to produce. Roughly speaking, the production of a country *does the maths* to synthetically encode the information about the endowments, our job consists in decoding this information. Unfortunately in the case of infrastructures we do not have such a scenario, we do not have a proxy which is the final output of the infrastructure stock. We can anyway think to define an infrastructure metrics by building an *educated* combination of a set of economic indicators related to infrastructures with the final aim to define a single figure per country. Examples of this strategy are the *Connectivity index, HDI, WEF-GCI Infrastructure pillar*. However, all these indicators suffer from a common methodological issue, they are typically subjective linearly weighted combination of survey-based scores and economic variables. In addition the weighting schemes are often updated (e.g., GCI pillars) year by year or even worse they are country/region dependent and this, generally, does not allow for consistent analysis over time.

Here instead we want to build a linear combination of variables related to the infrastructures with weights which are defined by the correlation structure among the variables themselves. In such a way we can capture as much as possible information with a single indicator. As in the case of Economic Fitness where we do not subjectively combine the country capabilities to define the fitness metrics, here we do not subjectively combine the indicators. We instead choose the weights as those maximizing the fraction of explained variance of our 47-dimensional dataset (the number of dimensions is the number of indicators we use). The statistical technique to achieve this is the *Principal Component Analysis* (PCA), a longstanding technique of dimensionality reduction which has been applied in several domains ranging from genetics [38] to finance [39] and machine learning. The PCA is essentially providing a new orthogonal basis to express the original set of data where the basis vectors (the components in the jargon of the PCA) are ordered by decreasing fraction of the original variance of the data. If the structure is not random, the PCA will find the preferential directions in the original dataset carrying a large fraction of the information and therefore, with a limited number of components (i.e., of dimensions), we will be essentially able to encode most of the information of the initial data. In addition, differently to more recent or complex techniques of dimensionality reduction [40], we do not lose the economic interpretability of the resulting indicator because we can directly inspect the loadings on the original variables for each component. We refer to [16,17] for a more formal introduction to PCA.

**Figure 1:**
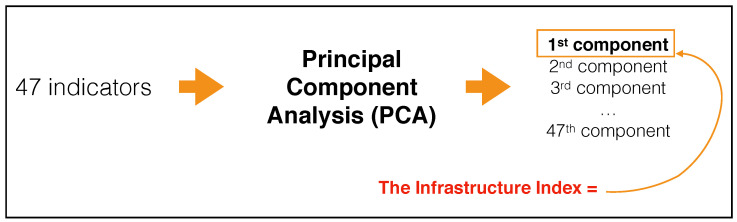
The proposed Infrastructure Index is the principal component issuing from a Principal Component Analysis performed on the 47 infrastructure indicators published by the World Bank. The indicator is a weighted linear combination as other synthetic infrastructure indicators but, differently from these other approaches, the weights are not set on a subjective basis but they are defined by the correlation structure of the data and they are optimal in terms of explained information.

In Figure 1, we show a flowchart summarizing the procedure we use to build the Infrastructure Index that we propose and leverage in this work.

In this section, we briefly discuss the main features of the components issuing from the PCA in relation to the other indicators we use in this work. An important preliminary step to perform a PCA analysis consists of choosing a policy to fill missing data and to scale/normalize the variables. The most common prescription to scale the variables for a PCA is to set all variables to have zero mean and unit variance. However, the 47 indicators we have are extremely heterogeneous in terms of nature (extensive and intensive), type (continuous and digitized) and span. Therefore we prefer to first perform a logarithmic transformation of all indicators and then set to zero mean the log-transformed variables. We do not rescale them to have unit variance (after the log transform the span and therefore the variance of the variables is significantly more homogeneous) because the fraction of explained variance of the first component is slightly higher without the unit variance rescaling (37% vs. 33%). Concerning the missing data, we set them to 0, i.e., the mean value after the rescaling. We refer to Appendix B for a detailed discussion on the normalization of the infrastructure variables and the general robustness of the results regardless of the alternative data sanitation policies we tested.

**Figure 2:**
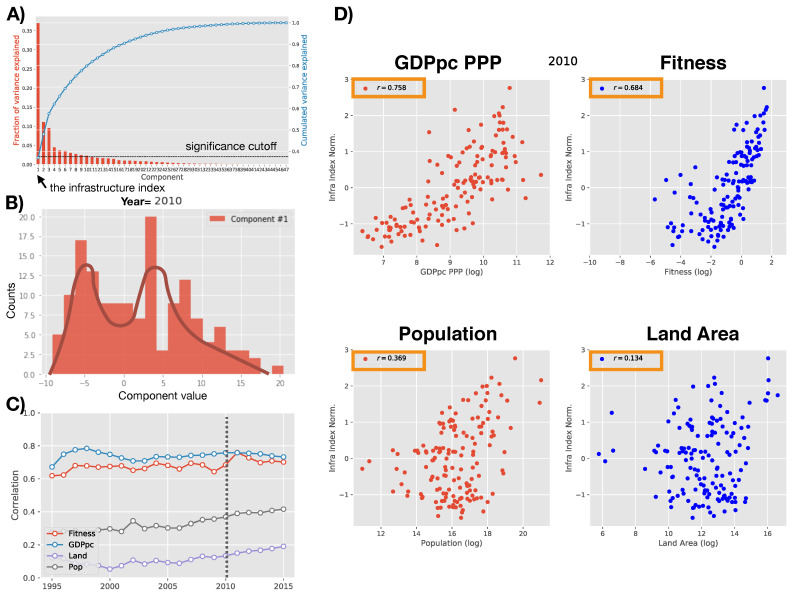
(Panel **A**): Fraction of variance per component. Components after the fifth explain a fraction of variance lower than the fraction expected if the system would be completely random (in that case, each component would carry approximately 1/47 of the total variance). The first component–our infrastructure indicator–accounts for approximately 37% of the variance, i.e., twenty times more than the significance cutoff. (Panel **B**): for illustrative purposes, we show the the cross-section distribution of the first component (non-standardized) in 2010 for the 149 countries we analyze. Our infrastructure index is the component #1 standardized to have 0-mean and unit variance cross-sectionally. (Panel **C**,**D**): The principal component (PC) is marginally correlated with population and country land area although an increasing trend is observed from 1995 to 2015. It is instead correlated with Fitness and GDPpc PPP (PPP=Power Parity Purchase) as expected (corr. = 0.7–0.8). In such a scenario deviations around the average behavior and the dynamics are the important playgrounds to understand the mutual relationship of these figures. Panel **D** illustrates the scatter plots from which the scores in panel **C** in 2010 are measured.

In panel A of Figure 2, we show the fraction of variance explained by each component. As we can see, the first one–our new infrastructure indicator–encodes approximately 37% of the original variance. We also see that starting from the 5th-6th component the variance explained starts to be compatible with the fraction expected if all variables would be random and uncorrelated. In Appendix B.3, we show the loadings of the first component on the original indicators. As we can see it is essentially a weighted average where all weights are positive (except for those indicators which correspond to a negative feature and the weights are consequently negative). In this perspective the first component is a clearly interpretable indicator. Starting from the second, the economic interpretability is less clear but as we will see those components are not connected to the growth and development of countries (see also Appendix B.5). In panel B of Figure 2, we illustrate the distribution in 2010 of the values in the direction of the first component for the 149 countries we consider. In panel C and D, we show the correlation of the first component each year with the GDPpc, the Economic Fitness, the population and the land area of countries. As expected the correlation is marginal with the latter two, while it is correlated with the Fitness and the GDPpc. This suggests that to understand the interplay between these three dimensions, as done in [19], we must look at their dynamic features as provided by their mutual time evolution.

## 3. Results

### 3.1. The Fitness-Infrastructure Dynamics

**Figure 3:**
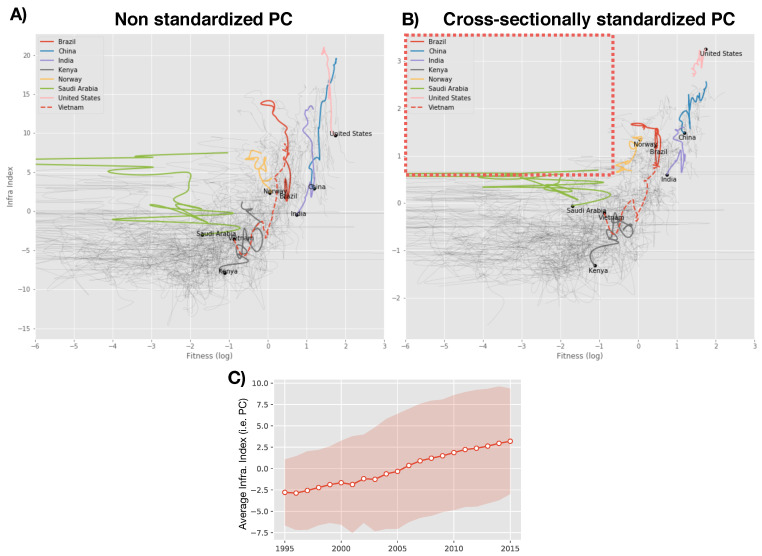
(Panel **A**): The Fitness-Infrastructure dynamics for the non-cross sectionally standardized principal component. (Panel **B**): same graph for the cross sectionally standardized principal component. The empty top left corner means that the level of infrastructures is inherently linked with the level of complexity of an economy. We do not observe countries with low fitness and with high infrastructure level while, conversely, we can observe countries with low fitness and high GDPpc countries because, at least on the short-mid term, the fitness of a country can be exchanged with exogenous rents as from natural resources. (Panel **C**): we show the temporal component of the infrastructure indicator we have previously defined by plotting the cross-section mean of the scores for the 149 countries in the direction of the PC. This drift corresponds to an increasing global level of infrastructures in this direction. The band corresponds to the cross-section standard dispersion of these scores.

As shown in [19], the evolution and interactions of economic variables can be effectively characterized in a dynamical space where the dimensions are defined by the variables themselves. In Figure 3, we show the trajectories of evolution in the space defined by the Economic Fitness and our infrastructure metrics from 1995 to 2015. We observe that the first component issuing from the PCA has a temporal (increasing) drift which can be interpreted as a global increasing level of infrastructures in the direction of this component (panel C of Figure 3). The Economic Fitness is a metrics which has a constant mean (see [23,24]). We then cross-sectionally normalize our infrastructure indicators (zero mean and unit variance) in order to be:

1. coherent with the Fitness metrics, in such a way both variables do not have a drift;
2. to naturally allow for time comparison.

For the sake of completeness, we show the dynamics with the non-standardized infrastructure index and the standardized one (Panel A and B respectively in Figure 3). In the following, we will discuss all the results with the standardized indicator but most of the results do not strongly depend on the specific version of the indicator. We highlight the trajectory for some selected countries and the general features, except for a general drift, are qualitatively unchanged. The highlighted countries allow us to briefly discuss some case studies which pinpoint the potential of this approach. We see that the trajectories of mature emerging countries as India and China are trailing the trajectory of developed countries as the United States. We also show the development of Vietnam which is one of the new emerging Asian countries which is essentially mimicking the path of India and Brazil in the last two decades. In [41] the analysis of growth scenarios for sub-Saharan countries in the Economic-Fitness-GDPpc plane suggests that Kenya is likely to be on the verge to start a sustained growth as Vietnam and other Asian countries did in the last decades. Interestingly the inclusion of the infrastructure dimension confirms and supports this scenario, Kenya is today very close to Vietnam position in the early nineties and may undergo the same development and be one of the first *frontier* African countries to be fully *emerging* in the next years.

The highlighted empty top left corner of Figure 3 (panel A or B) provides a first economic finding. If the same graph is drawn for the Economic Fitness vs. the GDPpc (see for instance [19]), we find that countries are spread in all the plane, and there exist countries with low fitness but high GDPpc. As discussed in [19,20,42], this is interpreted by the fact that the level of GDPpc can be explained with dimensions which are exogenous to the Fitness, typically natural resources rents (This is out of the scope of this work but the substitution of Fitness with exogenous rents tend to produce patterns of growth less predictable and more prone to negative shocks). The different behavior in the Fitness-Infrastructure plane means that, even though a country can fuel its income with exogenous sources as natural resources rents acting as a substitute for the Economic Fitness, the infrastructure level instead appears to be inherently linked with the complexity and diversity of a productive system.

**Figure 4:**
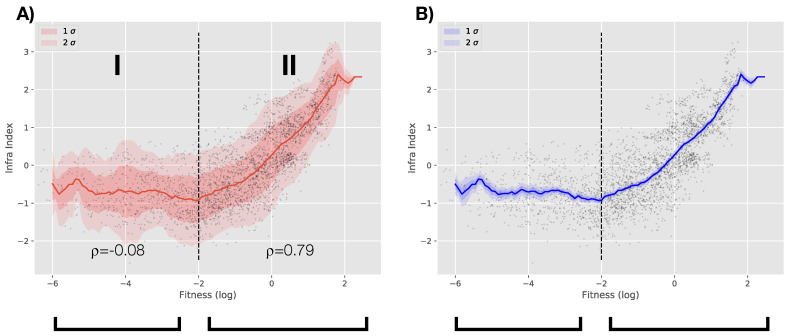
We run a rolling mean of the infrastructure level as a function of the Economic Fitness (width of the rolling window = 1). In the left panel (Panel **A**), red bands correspond to 1 and 2 standard deviation s while in the right panel (Panel **B**) we estimate the error of the rolling mean via a bootstrap. Two regimes emerge: regime I where the infrastructure level is on average constant regardless of the level of Fitness and mostly uncorrelated (ρ=−0.08); regime II where the two dimensions are significantly positively correlated (ρ=0.79).

In order to make this observation more quantitative, we perform a rolling mean of the infrastructure indicator for increasing value of the Fitness. This can be essentially thought as a local measure of the intensity of the relationship between the two. Results are shown in Figure 4. In Panel A, two regimes emerge. In the regime labeled *I*, the infrastructure level is constant regardless of the level of Fitness. In regime *II*, the Fitness and infrastructure level are approximately linearly related supporting the previous observation (the infrastructure level is inherently linked with the complexity and diversity of a productive system). In the left panel, the red band corresponds to 1 and 2-standard deviations while in the right panel, we bootstrap the events in each window of the rolling mean in order to test the stability of the mean (we are estimating the error of the rolling mean we are plotting). It is worth noticing that the threshold value of the Fitness (approx. at −2) separating the two regimes also corresponds to the transition from the chaotic to the laminar regime in the Fitness-GDPpc plane as discussed in [20]. This would suggest that infrastructures are directly linked with steady investment which are more likely to be achieved in a regime in which growth becomes more predictable and regular. For value of Fitness below this threshold, as show in [20], growth tends to be less predictable and extremely more volatile and, as shown here, in the same regime the level of infrastructure tends to be below the mean (the level is negative on average) and unrelated to the level of complexity of an economy. This would suggest that in the dynamic interplay between infrastructure and economic development, the latter plays a necessary role and sets a threshold which defines an infrastructure trap.

### 3.2. The Interplay between Economic Fitness and Infrastructures

Let us now focus on region II and deepen the investigation of the relationship between Fitness and infrastructure level, namely we want to understand if there is a preferred direction of interaction and if one of the variables leads more the development of the other one (or, alternatively, if the feedback is essentially symmetric).

There is no silver bullet to address this type of question. We then decide to devise an extremely simple and parsimonious setup to investigate the interplay. The question whether one variable leads the other can be mapped in measuring if there exists a lagged feedback of the infrastructure on the Economic Fitness and vice-versa and then compare strength and significance of the two specifications.

In order to do that, we investigate whether the Fitness *Granger*-causes (for the sake of simplicity, hereinafter, *causality* denotes *Granger*-causality) the infrastructure level and vice-versa up to a lag of 5 years (we refer to [22] for an introduction of the *Granger*-causality). The *Granger*-causality is a weaker (or rather a statistical) form of causality. The idea is that a time series *causes* a second time series if some lagged past values of the first time series are useful and retained (in a statistical way) in the augmented autoregression which includes past lagged value of both time series to forecast the current values of the second series.

In detail, we select the trajectories of those countries for which in 1995 (the first year for which we have the Fitness time series) the log(Fitness) is larger than −2 (we are essentially considering countries for which the starting point is in Regime II). In such a way, we retain 95 countries. For each country, we run two *causality* tests in order to measure whether Fitness *causes* the infrastructure index and whether the infrastructure index *causes* the Fitness.

A *Granger* analysis represents the simplest linear lagged relationship we can address to model the cause-effect relationship between two variables. In this perspective, it is a parsimonious setup. In fact, testing the *causality* corresponds to the assumption that the dynamical model specifying the interaction of the two variables is of the form:

(1) $$\begin{array}{cc} I_{t}^{c} & =\sum_{i=1}^{n}\alpha_{i}I_{t-i}^{c}+\sum_{j=1}^{m}\beta_{j}f_{t-j}^{c}+\epsilon_{t}\\ f_{t}^{c} & =\sum_{i=1}^{n}\gamma_{i}I_{t-i}^{c}+\sum_{j=1}^{m}\delta_{j}f_{t-j}^{c}+\epsilon_{t}^{\prime } \end{array}$$

where $f_{t}^{c}$ is the logarithm of the Fitness of country *c* at time *t* while $I_{t}^{c}$ is the infrastructure level of the same country and the time *t*. With the *Granger* test, we are essentially assessing if the terms corresponding to the $\beta_{i}$ and $\gamma_{i}$ coefficients are useful to forecast the value of the two indicators at time *t*. The analysis with lag 1 will include in the vector autoregressive model (VAR) of Equation (1) only the values one period behind, the analysis with lag 2 will include up to two periods behind, etc. We perform our analysis up to lag 5 years in order to have enough statistics.

**Figure 5:**
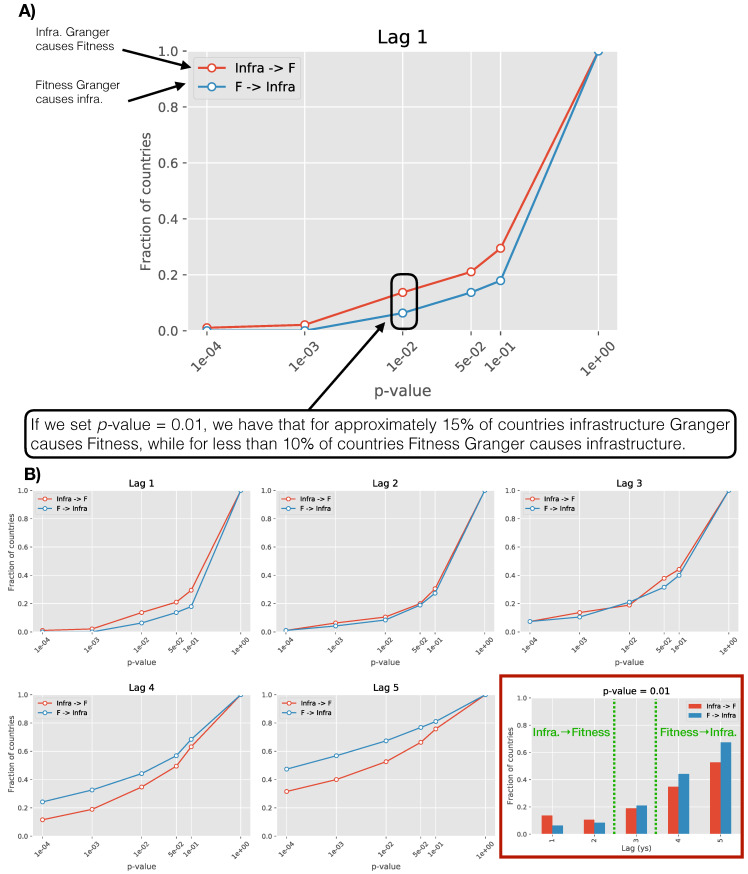
(Panel **A**): Guidelines to read results in panel **B**. For each lag, we run two *Granger* causality tests, one testing whether infrastructures *cause* Fitness (red) and one the reversed relation (blue). We then measure the fraction of countries for which the *causality* is validated as a function of the level of significance required for the validation. For instance, at a level of significance set by a *p*-value = 0.01, we have that infrastructure *causes* the Fitness in 15% of the 95 countries, while the vice-versa is only true in 1 out of 10 countries. If a line is consistently above the other, we conclude that there is a preferred way of interaction in the model specified in Equation (1) at that lag. (Panel **B**): The fraction of countries in which we observe *causality* for increasing value of the lag included in Equation (1). We see for small lags (<= 2 years) that infrastructure *causes* more the Fitness than the vice-versa while for increasing lags (>3 years) the relationship is reversed. In general the causality is validated for more countries when lags increase as shown in the last plot of Panel **B** (bottom-right graph). We plot the fraction of validated causality tests as a function of the lag for a specific value of the *p*-value, in this case *p*-value = 0.01.

In order to compare the two scenarios, for each lag we consider the fraction of countries for which the *causality* is validated (technically one minus the fraction for which the null-hypothesis is not rejected where the null-hypothesis is that the coefficients of the variables which is supposed to *cause* are 0 s). To show the consistence of our results with different thresholds of statistical validation (different values of *p*-values), we plot the above mentioned fraction of validated *Granger* events as a function of the *p*-value (i.e., statistical significance). We are therefore measuring the number of countries for which we find that the Fitness or the infrastructure level *causes* the infrastructure/Fitness with decreasing statistical significance. For infinite significance (*p*-value = 0) the fraction is obviously 0 while for no significance required the fraction is by construction 1. The results are shown in Figure 5. For each lag, we then have two curves, the line testing *Fitness causes infrastructure* (blue) and the one testing the *infrastructure causes Fitness* (red).

If one line is systematically above the second, we conclude that one direction of interplay is preferred, i.e., one variable tends to lead the second one. Interestingly, by increasing the lag, i.e., increasing the time horizon in Equation (1), the relationship appears to revert. We find that:

- **Lag 1–2 years**: infrastructure drives Fitness more than the opposite;
- **Lag 3 years**: contemporaneous;
- **Lag 4+ years**: Fitness *Granger*-pulls infrastructure significantly more than the opposite.

As a general trend, the fraction of validated results increases as the lag increases, indicating a general stronger effect for longer time horizon.

The inspection of the sign of the coefficients also provides insight if the effect is proactive or detrimental, and the results can be summarized as follows:

1. in region II for short time horizon (up 2 years) infrastructure appears to drive fitness evolution more than what Fitness does with infrastructures. As the coefficient of the VAR model are positive for almost all countries , we can say that infrastructures have a proactive role on Fitness on the short time horizon;
2. for longer time horizon the relationship is reversed, Fitness drives more infrastructure growth than the opposite (as for point 1, the coefficients’ sign tends to be positive, therefore the action is proactive);
3. the *Granger* causality is higher for longer time horizon, this means that, on the long term, the driving role of Fitness is more significant than the one of infrastructures on the short term;
4. in region I (not shown), results are less clear as the *Granger* test often does not converge within the level of tolerance set. However, in the perspective of the fact that in this region the infrastructure index is on average constant regardless of the value of the Fitness, we can conclude that Fitness and infrastructures has no evident mutual lagged effects as specified by Equation (1).

### 3.3. Do Countries Need Different Infrastructures in Different Development Regimes?

In the literature, it is often argued that countries in different stage of their development leverage or need different infrastructure stocks. Our framework permits to some extent to test this hypothesis. Figure 4 suggests that the Fitness induces a non-trivial ordering of countries with respect to the infrastructure stock. We run three different PCAs on four sets of countries on the basis of their Fitness: points with log(Fitness)<=−2 (regime I), points with −2<=log(Fitness)<=0 (Regime IIa) and points with log(Fitness)>0 (Regime IIb). The last two sets together correspond to regime II of the previous section which represents our fourth set for the PCA analysis.

**Figure 6:**
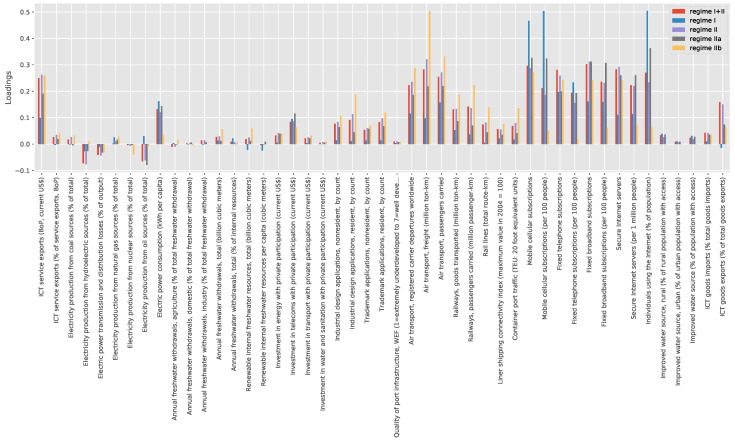
We plot the loadings of the principal component for different set of points of the Fitness-infrastructure plane conditioned on the value of Fitness. Regime I+II corresponds to all points available, Regime I is for log(Fitness)<=−2, Regime II is for log(Fitness)>−2, Regime IIa is for −2<=log(Fitness)<=0, Regime IIb is for log(Fitness)>0.

We compare the loadings of the principal component in Figure 6. The results are not completely conclusive as further analyses are required to understand whether the differences, especially between Regime I and IIb - blue and yellow bars, are significative. Unfortunately we cannot even replicate the Granger analysis on the two subsets of regime II because we would end up with too little statistics in order to draw reliable conclusions.

## 4. Discussion and Conclusions

This work represents an example to extend the paradigm of Economic Complexity beyond its initial scope introducing a new dimension which may affect the economic development of countries and their capability stock: the infrastructure stock. As for the Economic Fitness, a key point for this type of analysis is to develop a strategy to encapsulate in a low-dimensional indicator (possibly unidimensional) the economic dimension we want to measure.

To do this, we remain in the domain of linearly defined indicators but we propose a statistical procedure to define the optimal weights in terms of information to build a synthetic infrastructure index. This approach has two main advantages: (i) we maintain the economic interpretability of our indicator and (ii) at the same time, we devise the best possible indicator with respect to the information encoded in the set of indicators to combine.

With an indicator for infrastructures and for the complexity of the capability stock of a nation, we can investigate from a dynamic perspective the interplay between these two dimensions. By assuming a model of interplay as parsimonious as possible, i.e., a linear dependence on the past lagged values of both dimensions, we can quantify the degree of symmetry of the interplay by assessing if one dimension *Granger* causes the other one more than the opposite relation. As expected the two ways of interaction are at work but on the short term, the infrastructures are somehow the driving variables while on the long term the capability stock development leads the development of the infrastructures.

From a policy perspective, the existence of two regimes suggests that interventions in countries in *regime I* should predominately target the increase of the capability stocks, while in *regime II* policies should include interventions for both dimensions, on the short term for infrastructures and, on the long term, to strengthen the capability stock.

Concerning the perspectives we highlight some natural axes for future researches:

1. the *principal component analysis* is likely the simplest statistical technique to reduce the dimensionality of a space defined by several variables. We may envisage to explore more sophisticated techniques provided by current development of machine learning techniques but this would likely correspond to give up either the interpretability of the resulting metrics or the linearity;
2. the Fitness threshold at which the change of regime occurs is close to the level above which the growth of countries turns to be predictable and sustained while below economies tend to be subject to economic and political instabilities. It is worth investing the growth implication of this transition barrier and if the infrastructure indicator allows to extend or better define the regime of higher predictability for the growth found in [20,42].
3. in order to assess the dynamic interplay of infrastructures and Economic Fitness, we made the most parsimonious hypothesis for their interaction: a linear dynamic model depending only on the past values of the two dimensions via constant coefficients. However, we may think to extend the analysis either including non-linear terms or via more complex statistical techniques to assess the causality of time series [43,44,45].
4. the extension of the dynamical-system approach to study the interplay between infrastructure investments and the synthetic index we propose;
5. understand whether the differences in the infrastructure stock we observe have a statistical significance;
6. deepening the understanding of the components after the first by linking these components with the capability stock at sector level (i.e., the sector Economic Fitness).

## Figures and Tables

**Figure A1:**
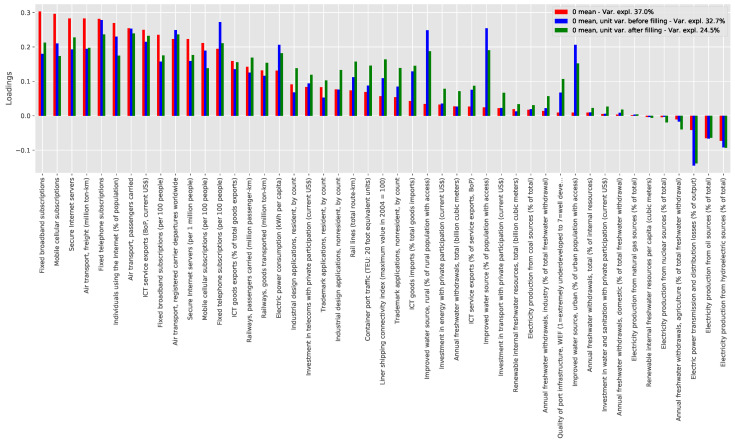
We show the loadings of the principal component for different policies to clean and fill missing values. Results are essentially left unchanged by different sanitation policies.

**Figure A2:**
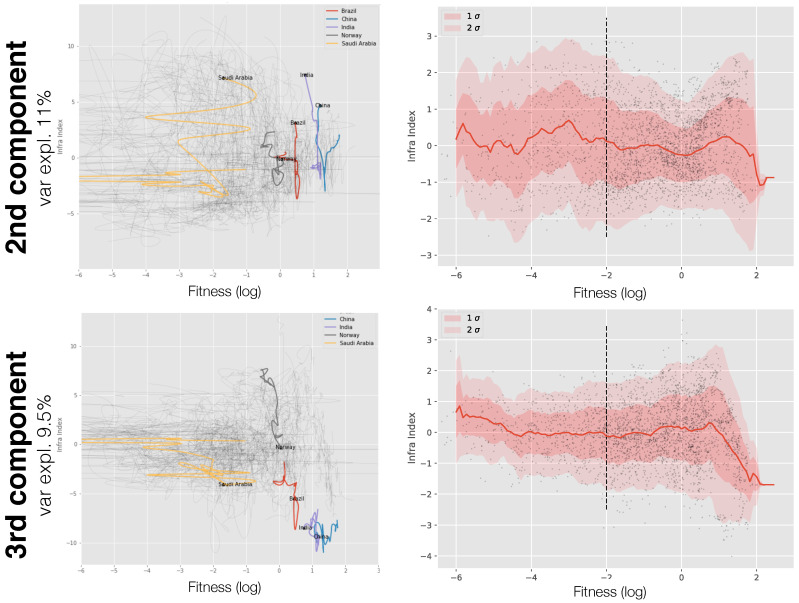
The Economic Fitness-infrastructure relation for the components from the second to the third. We remind that after the fifth, the amount of variance explained by the components is compatible with a random direction. Differently from the first component, no clear dependence emerges, the level of infrastructures is essentially constant regardless of the the Fitness value and the *Granger* causality analysis on the components after the first does not provide conclusive results.

**Figure A3:**
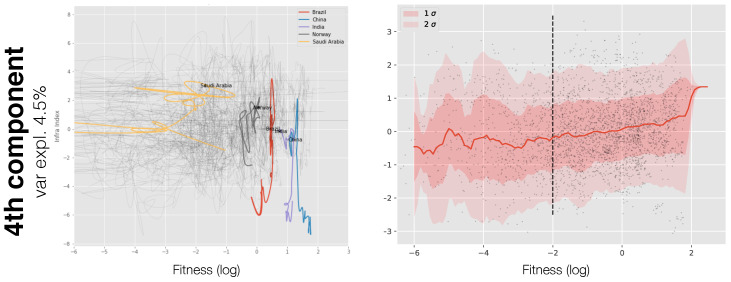

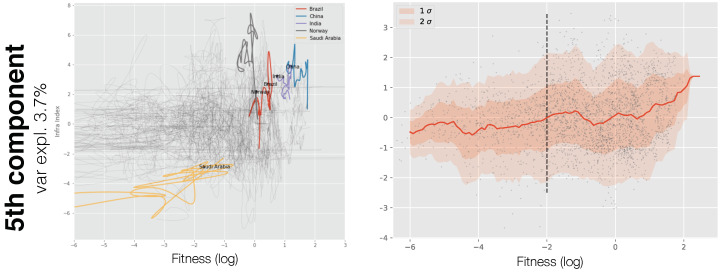
The Economic Fitness-infrastructure relation for the components from the fourth to the fifth. We remind that after the fifth, the amount of variance explained by the components is compatible with a random direction. Differently from the first component, no clear dependence emerges, the level of infrastructure is essentially constant regardless of the the Fitness value and the *Granger* causality analysis on the components after the first does not provide conclusive results.

## Appendix A. Data

### Appendix A.1. Bilateral Trade Data

The Economic Fitness metrics is the final output of an iterative algorithm leveraging the International Trade Statistics Database which is made publicly available by United Nations and accessible via UN Comtrade website [27]. In this specific work we use a *cleaned* version of the UN Comtrade raw data: the BACI data set released by CEPII. The BACI dataset is the result of reconciliation procedure of the UN Comtrade which essentially fixes the inconsistencies between import and export flows, see [28] for details. The release of the BACI data set we use spans the period 1995–2015. This data set provides all yearly trade volumes, expressed in current USD, between pairs of countries broken down at the product level. Products are available up to the 6-digit level of the Harmonized System classification. In this specific work, we will use products aggregated at 4-digit level (HS2007) to compute the Economic Fitness. At 4-digit level, we have approximately 1150 products exported by at least one country (we discard those products for which all trade flows are zero). Eventually we filter out countries according to a population and total export volume threshold. This procedure selects approximately 150 countries which account for more than 95% of world GDP.

### Appendix A.2. Economic Fitness

Alternatively, the Economic Fitness indicator has been recently released by World Bank and can be directly obtained at this link [26].

### Appendix A.3. Infrastructure Indicators

At the beginning of this work (spring 2017), the World Bank data portal provided 47 indicators under the section infrastructures. We use the indicator time series from 1995 to 2015 and we consider them for 149 countries. As a result, we end up with 3129 possible observations to feed the PCA. We report in the following table a short description of the 47 indicators. This work has been carried with the indicators as available in May 2017 [18].

**Table A1:** The 47 infrastructure indicators provided by the World Bank portal together with a short  descriptions.

	World Bank Code	Description
1	SH.H2O.SAFE.UR.ZS	Improved water source, urban (% of urban population with access)
2	EG.ELC.PETR.ZS	Electricity production from oil sources (% of total)
3	IT.NET.BBND.P2	Fixed broadband subscriptions (per 100 people)
4	ER.H2O.FWDM.ZS	Annual freshwater withdrawals, domestic (% of total freshwater withdrawal)
5	SH.H2O.SAFE.RU.ZS	Improved water source, rural (% of rural population with access)
6	IP.TMK.NRCT	Trademark applications, nonresident, by count
7	BX.GSR.CCIS.ZS	ICT service exports (% of service exports, BoP)
8	EG.ELC.NGAS.ZS	Electricity production from natural gas sources (% of total)
9	EG.ELC.NUCL.ZS	Electricity production from nuclear sources (% of total)
10	IT.CEL.SETS.P2	Mobile cellular subscriptions (per 100 people)
11	IT.NET.SECR	Secure Internet servers
12	IE.PPI.WATR.CD	Investment in water and sanitation with private participation (current US$)
13	IP.IDS.RSCT	Industrial design applications, resident, by count
14	IT.MLT.MAIN.P2	Fixed telephone subscriptions (per 100 people)
15	SH.H2O.SAFE.ZS	Improved water source (% of population with access)
16	ER.H2O.INTR.PC	Renewable internal freshwater resources per capita (cubic meters)
17	EG.ELC.COAL.ZS	Electricity production from coal sources (% of total)
18	EG.USE.ELEC.KH.PC	Electric power consumption (kWh per capita)
19	IQ.WEF.PORT.XQ	Quality of port infrastructure
20	ER.H2O.FWTL.ZS	Annual freshwater withdrawals, total (% of internal resources)
21	IS.AIR.PSGR	Air transport, passengers carried
22	ER.H2O.FWIN.ZS	Annual freshwater withdrawals, industry (% of total freshwater withdrawal)
23	IE.PPI.ENGY.CD	Investment in energy with private participation (current US$)
24	ER.H2O.FWAG.ZS	Annual freshwater withdrawals, agriculture (% of total freshwater withdrawal)
25	TM.VAL.ICTG.ZS.UN	ICT goods imports (% total goods imports)
26	EG.ELC.LOSS.ZS	Electric power transmission and distribution losses (% of output)
27	IT.MLT.MAIN	Fixed telephone subscriptions
28	IS.RRS.GOOD.MT.K6	Railways, goods transported (million ton-km)
29	ER.H2O.INTR.K3	Renewable internal freshwater resources, total (billion cubic meters)
30	TX.VAL.ICTG.ZS.UN	ICT goods exports (% of total goods exports)
31	IS.AIR.DPRT	Air transport, registered carrier departures worldwide
32	IS.RRS.TOTL.KM	Rail lines (total route-km)
33	IT.NET.BBND	Fixed broadband subscriptions
34	IT.CEL.SETS	Mobile cellular subscriptions
35	IS.AIR.GOOD.MT.K1	Air transport, freight (million ton-km)
36	BX.GSR.CCIS.CD	ICT service exports (BoP, current US$)
37	IT.NET.SECR.P6	Secure Internet servers (per 1 million people)
38	P.IDS.NRCT	Industrial design applications, nonresident, by count
39	IE.PPI.TRAN.CD	Investment in transport with private participation (current US$)
40	IT.NET.USER.ZS	Individuals using the Internet (% of population)
41	IP.TMK.RSCT	Trademark applications, resident, by count
42	EG.ELC.HYRO.ZS	Electricity production from hydroelectric sources (% of total)
43	IE.PPI.TELE.CD	Investment in telecoms with private participation (current US$)
44	ER.H2O.FWTL.K3	Annual freshwater withdrawals, total (billion cubic meters)
45	IS.SHP.GOOD.TU	Container port traffic (TEU: 20 foot equivalent units)
46	IS.RRS.PASG.KM	Railways, passengers carried (million passenger-km)
47	IS.SHP.GCNW.XQ	Liner shipping connectivity index (maximum value in 2004 = 100)

### Appendix A.4. Other Indicators

The source of all the remaining economic indicators used in this work–GDP, GDP *per capita*, population and country land area–is the World Bank Open Data platform [18]. This work has been carried with the indicators as available in May 2017.

## Appendix B. Principal Component Analysis

### Appendix B.1. Robustness

We discuss alternative definition or data sanitation procedures for the 47 indicators before performing the PCA.

In Figure A1 we show the results for the loadings for different normalization/missing data filling policies, namely:

- we center all indicators in order to have 0-mean (red);
- we center all indicators in order to have 0-mean (blue) and scale to have unit variance before filling missing data;
- we center all indicators in order to have 0-mean (green) and scale to have unit variance after filling missing data.

We observe that the composition of the PC is mostly independent on the specifications of the normalization. The specifications we used for our analysis are those ensuring the highest explained variance (i.e., case called 0-mean, red bars).

### Appendix B.2. Selecting the Variables

We also test less *agnostic* approaches by performing educated selections of infrastructure indicators to feed the PCA. In this way we can also verify that most of our results, at least on a qualitative basis, do not depend on the details of the selection of the indicators which we include in the statistical analysis.

In detail, we group the indicators in two major groups, the intensive indicators (*per capita* or per unit of GDP) and the extensive ones (those which instead scale with the size of the country/population or with the GDP). We have 24 indicators in the first group and 21 in the second one (two indicators are redundant or do not fall in these two categories).

We also consider the case with the complete set of 47 indicators in which we rescale the extensive ones with the country population in order to have only intensive variables as a input of the PCA.

We find that:

- the variance explained by the first component is enhanced when we separate the analysis, 49% for extensive and 40% for intensive variables. The variance is instead left essentially unchanged in the third case mentioned–all indicators but the extensive ones are rescaled to be *per capita*, i.e., 38%.
- more importantly, on a qualitative basis, the results concerning the Granger causality between Economic Fitness and infrastructures are essentially left unchanged regardless of the subset of variables we select.
- The inversion of the driving role between Economic Fitness and infrastructure at 3 years is clearer when the extensive variables are considered or included in the PCA specifications.

### Appendix B.3. Interpreting the Loadings of the Principal Component

Figure A1 allows us to interpret the economic meaning of our infrastructure indicator. First of all, all the weights are positive (most of the negative loadings correspond to variables with a negative sense). This means that our indicator is, conceptually speaking, an indicator belonging to the class of linear combinations of pillars as the GCI. The few apparent anomalies are related to negative loadings on some indicators of electricity production from specific sources as from oil. However, the indicator corresponding to electricity availability is strongly positive. Interestingly the loadings suggest a scenario where the electricity availability matters more than the production to quantify country infrastructures.

Interestingly, the same observation holds for investment as well. We find indeed that loadings on those indicators corresponding to investments measured in USD tend to have small loadings suggesting that these directions are less informative to capture the infrastructure stock of a country. From an economic point of view, this suggests, as for electricity, that the outcome of infrastructures is more important that the amount invested measured in USD.

### Appendix B.4. The Loadings of the Principal Component

We report in Table the loadings, sorted by decreasing weight, defining the Infrastructure Index used in this work (red bars of Figure A1).

It is also worth noticing that ICT service exports have one of the largest loadings and from [35,46] we also know they are also in the complex section of the progression network (a network measuring the most recurrent trajectories of development product wise). Our results support the importance of those services which are simultaneously a high value added export and one of the major contributions to our Infrastructure Index.

**Table A2:** The loadings of the Infrastructure Index on the original 47 indicators. We highlight the indicators measuring the direct investment in USD. They are all positive confirming the results in [7,9,10,11,12] but interestingly the loadings tend to be small on investment indicators. This suggests that the investment dimension alone is less informative than expected to measure the infrastructure stock.

Description	Loading
Fixed broadband subscriptions	0.303
Mobile cellular subscriptions	0.296
Secure Internet servers	0.283
Air transport, freight (million ton-km)	0.283
Fixed telephone subscriptions	0.281
Individuals using the Internet (% of population)	0.270
Air transport, passengers carried	0.255
ICT service exports (BoP, current US$)	0.250
Fixed broadband subscriptions (per 100 people)	0.235
Air transport, registered carrier departures worldwide	0.223
Secure Internet servers (per 1 million people)	0.223
Mobile cellular subscriptions (per 100 people)	0.212
Fixed telephone subscriptions (per 100 people)	0.195
ICT goods exports (% of total goods exports)	0.159
Railways, passengers carried (million passenger-km)	0.142
Railways, goods transported (million ton-km)	0.132
Electric power consumption (kWh per capita)	0.131
Industrial design applications, resident, by count	0.091
**Investment in telecoms with private participation (current US$)**	0.084
Trademark applications, resident, by count	0.083
Industrial design applications, nonresident, by count	0.077
Rail lines (total route-km)	0.074
Container port traffic (TEU: 20 foot equivalent units)	0.069
Liner shipping connectivity index (maximum value in 2004 = 100)	0.057
Trademark applications, nonresident, by count	0.054
ICT goods imports (% total goods imports)	0.043
Improved water source, rural (% of rural population with access)	0.034
**Investment in energy with private participation (current US$)**	0.033
Annual freshwater withdrawals, total (billion cubic meters)	0.027
ICT service exports (% of service exports, BoP)	0.027
Improved water source (% of population with access)	0.025
**Investment in transport with private participation (current US$)**	0.022
Renewable internal freshwater resources, total (billion cubic meters)	0.019
Electricity production from coal sources (% of total)	0.017
Annual freshwater withdrawals, industry (% of total freshwater withdrawal)	0.014
Quality of port infrastructure, WEF (1 = extremely underdeveloped to 7 = well deve...	0.010
Improved water source, urban (% of urban population with access)	0.009
Annual freshwater withdrawals, total (% of internal resources)	0.009
**Investment in water and sanitation with private participation (current US$)**	0.005
Annual freshwater withdrawals, domestic (% of total freshwater withdrawal)	0.003
Electricity production from natural gas sources (% of total)	0.002
Renewable internal freshwater resources per capita (cubic meters)	−0.004
Electricity production from nuclear sources (% of total)	−0.004
Annual freshwater withdrawals, agriculture (% of total freshwater withdrawal)	−0.010
Electric power transmission and distribution losses (% of output)	−0.041
Electricity production from oil sources (% of total)	−0.065
Electricity production from hydroelectric sources (% of total)	-0.073

### Appendix B.5. Components after the First

In Figure A2 and Figure A3, we show the dynamics in the Fitness-n−th component for n=2,3,4,5. Differently from the first component, we do not find any clear and robust relation between the Fitness and the infrastructures as measured by successive components. This does not necessarily imply that these components do not affect the economic development, the interaction might be non-linear or only on specific sectors/countries. The understanding of the role of these components is beyond the scope of this work and will be investigated in future works.

## References

[1] Aschauer D.A. (1989). Is public expenditure productive?. J. Monet. Econ..

[2] Romp W., de Haan J. (2007). Public capital and economic growth: A critical survey. Perspekt. Wirtsch..

[3] Bom P., Ligthart J.E. (2008). How Productive is Public Capital? A Meta-Analysis. https://ssrn.com/abstract=1088651.

[4] Straub S. (2008). Infrastructure and Development: A Critical Appraisal of the Macro Level Literature.

[5] Calderón C., Moral-Benito E., Servén L. (2011). Is Infrastructure Capital Productive? A Dynamic Heterogeneous Approach.

[6] Estache A., Wren-Lewis L. (2009). Toward a theory of regulation for developing countries: Following jean-jacques laffont’s lead. J. Econ. Lit..

[7] Garsous G. (2012). How Productive is Infrastructure? A Quantitative Survey.

[8] Fernald J.G. (1999). Roads to prosperity? Assessing the link between public capital and productivity. Am. Econ. Rev..

[9] Binswanger H.P., Khandker S.R., Rosenzweig M.R. (1993). How infrastructure and financial institutions affect agricultural output and investment in India. J. Dev. Econ..

[10] Estache A., Speciale B., Veredas D. (2005). How Much Does Infrastructure Matter to Growth in Sub-Saharan Africa?.

[11] Qiang C.Z.W., Pitt A. (2003). Contribution of Information and Communication Technologies to Growth.

[12] Chakraborty C., Nandi B. (2011). ‘Mainline’ telecommunications infrastructure, levels of development and economic growth: Evidence from a panel of developing countries. Telecommun. Policy.

[13] Buys P., Deichmann U., Wheeler D. (2006). Road Network Upgrading and Overland Trade Expansion in Sub-Saharan Africa.

[14] Wilson J., Mann C., Otsuki T. (2003). Trade Facilitation and Economic Development: Measuring the Impact.

[15] Estache A., Garsous G. (2012). The impact of infrastructure on growth in developing countries. IFC Econ. Notes.

[16] Shlens J. (2014). A tutorial on principal component analysis. arXiv.

[17] Jolliffe I.T. (2002). Graphical representation of data using principal components. Princ. Compon. Anal..

[18] World Bank Data Portal. https://data.worldbank.org/indicator?tab=all.

[19] Cristelli M., Tacchella A., Pietronero L. (2015). The heterogeneous dynamics of economic complexity. PLoS ONE.

[20] Cristelli M., Tacchella A., Cader M., Roster K., Pietronero L. (2017). On the Predictability of Growth.

[21] Tacchella A., Mazzilli D., Pietronero L. (2018). A dynamical systems approach to gross domestic product forecasting. Nat. Phys..

[22] Granger C.W. (1969). Investigating causal relations by econometric models and cross-spectral methods. Econ. J. Econ. Soc..

[23] Cristelli M., Gabrielli A., Tacchella A., Caldarelli G., Pietronero L. (2013). Measuring the intangibles: A metrics for the economic complexity of countries and products. PLoS ONE.

[24] Tacchella A., Cristelli M., Caldarelli G., Gabrielli A., Pietronero L. (2012). A new metrics for countries’ fitness and products’ complexity. Sci. Rep..

[25] Servedio V.D., Buttà P., Mazzilli D., Tacchella A., Pietronero L. (2018). A new and stable algorithm for economic complexity. arXiv.

[26] Economic Fitness. https://datacatalog.worldbank.org/dataset/economic-fitness.

[27] Un COMTRADE Portal. https://comtrade.un.org.

[28] Gaulier G., Zignago S. (2010). Baci: International Trade Database at the Product-Level (the 1994–2007 Version). https://ssrn.com/abstract=1994500.

[29] Mariani M.S., Vidmer A., Medo M., Zhang Y.C. (2015). Measuring economic complexity of countries and products: Which metric to use?. Eur. Phys. J. B.

[30] Wu R.J., Shi G.Y., Zhang Y.C., Mariani M.S. (2016). The mathematics of non-linear metrics for nested networks. Phys. A Stat. Mech. Appl..

[31] Pietronero L., Cristelli M., Gabrielli A., Mazzilli D., Pugliese E., Tacchella A., Zaccaria A. (2017). Economic Complexity: Buttarla in caciara vs a constructive approach. arXiv.

[32] Gabrielli A., Cristelli M., Mazzilli D., Tacchella A., Zaccaria A., Pietronero L. (2017). Why we like the ECI+ algorithm. arXiv.

[33] Hidalgo C.A., Hausmann R. (2009). The building blocks of economic complexity. Proc. Natl. Acad. Sci. USA.

[34] Stojkoski V., Utkovski Z., Kocarev L. (2016). The impact of services on economic complexity: Service sophistication as route for economic growth. PLoS ONE.

[35] Zaccaria A., Mishra S., Cader M.Z., Pietronero L. (2018). Integrating Services in the Economic Fitness Approach.

[36] Angelini O., di Matteo T. (2018). Complexity of products: The effect of data regularisation. arXiv.

[37] Dosi G., Nelson R., Winter S. (2001). The Nature and Dynamics of Organizational Capabilities.

[38] Price A.L., Patterson N.J., Plenge R.M., Weinblatt M.E., Shadick N.A., Reich D. (2006). Principal components analysis corrects for stratification in genome-wide association studies. Nat. Genet..

[39] Laloux L., Cizeau P., Potters M., Bouchaud J.P. (2000). Random matrix theory and financial correlations. Int. J. Theor. Appl. Financ..

[40] Kohonen T. (1982). Self-organized formation of topologically correct feature maps. Biol. Cybern..

[41] Cristelli M., Tacchella A., Zaccaria A., Pietronero L. (2014). Growth Scenarios for Sub-Saharan Countries in the Framework of Economic Complexitya. https://mpra.ub.uni-muenchen.de/id/eprint/71594.

[42] Pugliese E., Chiarotti G.L., Zaccaria A., Pietronero L. (2017). Complex economies have a lateral escape from the poverty trap. PLoS ONE.

[43] Sugihara G., May R., Ye H., Hsieh C.H., Deyle E., Fogarty M., Munch S. (2012). Detecting causality in complex ecosystems. Science.

[44] Brunton S.L., Proctor J.L., Kutz J.N. (2016). Discovering governing equations from data by sparse identification of nonlinear dynamical systems. Proc. Natl. Acad. Sci. USA.

[45] Pierce D.A., Haugh L.D. (1977). Causality in temporal systems: Characterization and a survey. J. Econ..

[46] Zaccaria A., Cristelli M., Tacchella A., Pietronero L. (2014). How the taxonomy of products drives the economic development of countries. PLoS ONE.

